# Bioactive Compounds from Marine Sponges and Algae: Effects on Cancer Cell Metabolome and Chemical Structures

**DOI:** 10.3390/ijms231810680

**Published:** 2022-09-14

**Authors:** Roberta Esposito, Serena Federico, Francesca Glaviano, Emanuele Somma, Valerio Zupo, Maria Costantini

**Affiliations:** 1 Stazione Zoologica Anton Dohrn, Department of Ecosustainable Marine Biotechnology, Via Ammiraglio Ferdinando Acton, 80133 Napoli, Italy; 2 Department of Biology, University of Naples Federico II, Complesso Universitario di Monte Sant’Angelo, Via Cinthia 21, 80126 Napoli, Italy; 3 Stazione Zoologica Anton Dohrn, Department of Ecosustainable Marine Biotechnology, Ischia Marine Centre, 80077 Ischia, Italy; 4 Department of Life Sciences, University of Trieste, 34127 Trieste, Italy

**Keywords:** algae, cancer, sponges, marine eukaryotes, metabolism

## Abstract

Metabolomics represent the set of small organic molecules generally called metabolites, which are located within cells, tissues or organisms. This new “omic” technology, together with other similar technologies (genomics, transcriptomics and proteomics) is becoming a widely used tool in cancer research, aiming at the understanding of global biology systems in their physiologic or altered conditions. Cancer is among the most alarming human diseases and it causes a considerable number of deaths each year. Cancer research is one of the most important fields in life sciences. In fact, several scientific advances have been made in recent years, aiming to illuminate the metabolism of cancer cells, which is different from that of healthy cells, as suggested by Otto Warburg in the 1950s. Studies on sponges and algae revealed that these organisms are the main sources of the marine bioactive compounds involved in drug discovery for cancer treatment and prevention. In this review, we analyzed these two promising groups of marine organisms to focus on new metabolomics approaches for the study of metabolic changes in cancer cell lines treated with chemical extracts from sponges and algae, and for the classification of the chemical structures of bioactive compounds that may potentially prove useful for specific biotechnological applications.

## 1. Introduction: A New “Omics” Technology: Metabolomics

**Figure 1:**
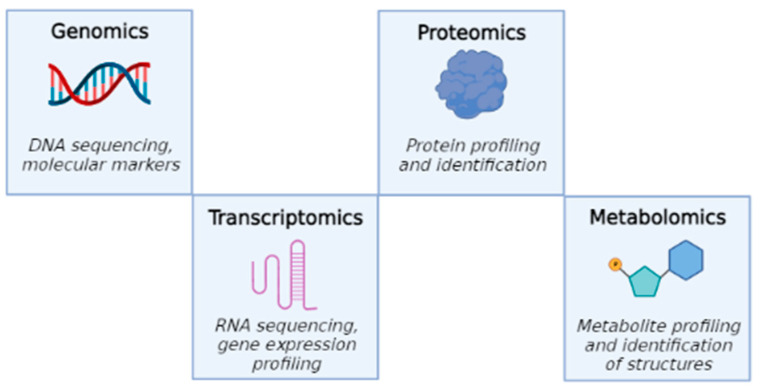
Four major “omics” fields, starting from genomics to metabolomics.

Metabolomics (or metabonomics [1]) is used to define the large-scale study of small organic molecules, commonly known as metabolites (small molecules of 1 kilodalton, KDa), present within cells, biofluids, tissues or organisms. All of these small molecules taken together, and their interactions within a biological system, are known as the metabolome. This new discipline, together with the other omics techniques, helps reaching a complete view of cellular processes. The aim of this approach is to sketch, in cooperation with other omics technologies (genomics, transcriptomics and proteomics), a network of interactions that can describe, in detail, the state of the cell. This is called “interactome analysis” [2]. In fact, while genomics is the study of DNA and genetic information within a cell, transcriptomics is the study of RNA and differences in mRNA expression; proteomics is the large-scale study of proteins produced in an organism, system or biological context. In turn, metabolomics completes these studies, analyzing the substrates and products of metabolism as they are influenced by genetic and environmental factors (Figure 1).

**Figure 2:**
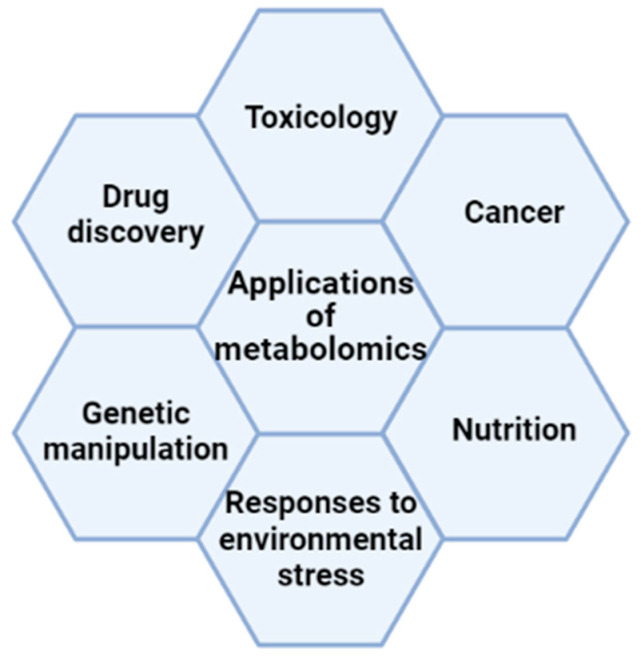
The main applications of metabolomics and their relationships.

Metabolomics represents a powerful approach, because metabolites and their concentrations directly reflect the underlying biochemical activity and state of cells and/or tissues, producing the molecular phenotype. The set of these small molecules, such as peptides, amino acids, nucleic acids, carbohydrates, organic acids, vitamins, polyphenols and alkaloids, represents the metabolome, through which the final or intermediate products of a biochemical process can be studied in order to build a metabolic pathway [2]. This area of research produces a “photo” of a cell, which can help identify its current phase in the cell cycle, and help determine whether or not it is facing environmental stress or if it is correctly performing its physiological role [3]. In fact, metabolomics approaches are extensively used i. to evaluate responses to environmental stress; ii. to investigate toxicology, drug discovery [4] and nutrition [5]; iii. to study the global effects of genetic manipulation and cancer; iv. to discover natural products; and v. to compare different stages of growth [6,7,8,9,10,11] (Figure 2).

**Figure 3:**
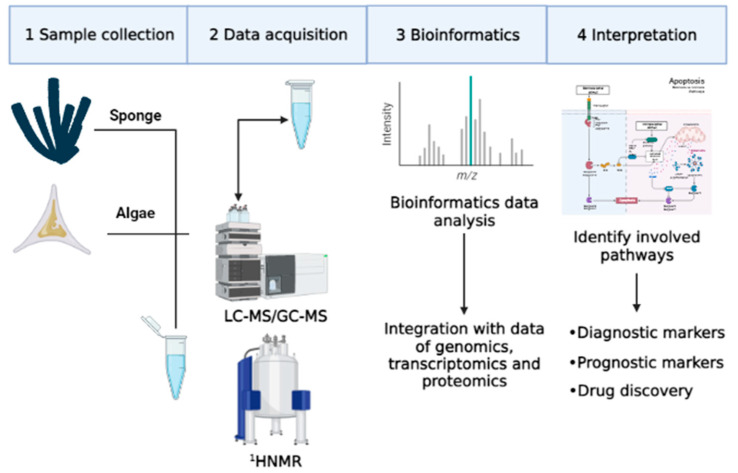
Main steps of metabolomics technologies.

The concept of the metabolic profile first appeared in literature in the 1950s, but only in the following three decades was this area of research completely developed. Despite this observation, metabolomics has only recently aroused the interest of researchers, thanks to the development of advanced technologies for the quantification of metabolites, such as gas chromatography (GC) and mass spectrometry (MS) [3]. Initially, studies mainly concerned metabolites of specific compounds such as pharmaceutical products [12,13]. Later, in this research area, studies on classes of compounds were included, such as catecholamines and oxylipins [14,15,16,17]. In particular, quantitative determination of some metabolites of catecholamines was performed with metabolomics approaches. These metabolites were used as biological markers for diagnosis, for evaluation of therapeutic responses, and for early recognition of tumor relapses derived from the neural crest (neuroblastoma, pheochromocytoma), carcinoid tumors and melanoma [14]. In addition, metabolomics approaches were used to identify oxygenated fatty acid derivatives, called oxylipins, mainly produced from diatoms. Oxylipins have a negative effect on reproduction and on the development of different marine invertebrates, such as copepods [18] and sea urchins [19], but these molecules also have a cytotoxic effect on several cancer cell lines [20]. Metabolomic research is primarily carried out in complex matrices such as blood, cells, plants or extracts of other marine organisms [21]. Therefore, appropriate sample preparation and analysis techniques are necessary for a rapid and simultaneous determination of various compounds (Figure 3) [22].

## 2. Main Metabolomics Methodologies

Different methods can be applied to prepare, to extract and to analyse samples. In this paragraph, we explain the most used techniques to treat marine specimens in order to avoid rapid alteration of their metabolic profile and to stop metabolic reactions.

### 2.1. Sample and Extraction Techniques

Obtaining a broad coverage of the metabolome is difficult, due to a wide range of physico-chemical properties exhibited by small molecules. For this reason, various techniques are used to evaluate the set of metabolites [23]. Once samples are collected, it is recommended to treat them with liquid nitrogen or specific solvents [24,25,26]. Since extracts, for example, from marine organisms, contain a high percentage of salt and lipids that interfere with the most common analytical methods used in metabolomics, such as LC-MS and HPLC, they must be eliminated. The most used methods for this purpose are solid-phase extraction with Diaion HP-20 Resins, pre-equilibrated with methanol [27], or C18 and PS-DVB SPE cartridges [28,29], Sephadex LH-20 with a mobile phase of methanol and dichloromethane (1:1) or C18 SPE cartridges that are highly lipophilic [27]. After sample collection and preparation, separation techniques, such as liquid chromatography (LC) in its high performance (HPLC) or ultra-performance (UPLC) forms, gas chromatography (GC) and detection techniques such as mass spectrometry (MS) and nuclear magnetic resonance (NMR) are used [23,24,25]. Further, acquired data are processed in order to create a numerical matrix, which can be used for statistical or multivariate analysis [25]. Generally, the treatment of Nuclear Magnetic Resonance (NMR) data is simpler than that required for LC-MS data, consisting of phase correction; baseline adjustment; shift adjustment and binning that divides an NMR spectrum into many regions or bins to reduce the effects of pH, composition and ionic strength of sample [30]; fuzzy warping (an algorithm that can be used to establish correspondence between the most intense peaks of the spectra to be aligned) [31]; peak alignment using a genetic algorithm [32]; and normalization [33]. For LC–MS data, different software are available for data handling, some of which are open access, such as OpenMS [34], MZMine 2 [35], XCMS [36] and MS-DIAL [37]. *t*-test, analysis of variance, principal component analysis (PCA), and partial least squares (PLS) and orthogonal-PLS (OPLS) analyses are the most used statistical methods in metabolomics studies [38,39]. In the following paragraphs, the most important separation and detection techniques are summarized.

### 2.2. Separation Techniques

Several separation techniques are routinely applied according to the characteristics of the putative compounds to be identified. Among them, Gas Chromatography (GC) is an excellent separation technique that was considerably improved with the introduction in 1979 of fused-silica capillary columns that resulted in higher resolution, higher efficiency, better reproducibility and smaller sample size [40]. In addition, the combination of gas chromatography with a mass spectrometer may be a highly sensitive approach. However, GC is limited to small compounds, which are thermally stable, volatile or can be rendered chemically volatile, for instance, by trimethylation [41]. Detection in GC analysis may be limited to certain compounds unless Mass Spectrometry (MS) is the method of choice, however, even when MS is used, some of the compounds may not be ionized sufficiently to be detected at low levels unless they are derivatized with an ionisable moiety [42]. HPLC and UHPLC are powerful tools for metabolomic studies that enable the separation and characterization of metabolite similarities. These techniques represent efficient separation technologies, which can be used to determine different groups of compounds, hydrophilic as well as hydrophobic, salts, acids, bases, etc. [6]. HPLC, unlike GC, is not limited to the separation of thermally stable volatile compounds or large molecules. The separation of each group within the HPLC is a function of the solute properties that determine the column type (stationary phase) and mobile phase to be used for successful separation [43]. These modes include RP (reverse phase), normal phase, ion exchange, chiral, size exclusion, hydrophilic interaction chromatography (HILIC) and mixed modes [42,44].

### 2.3. Detection Techniques

MS and NMR are the most widely used analytical techniques in metabolomics [45]. MS provides a mix of rapid, sensitive and selective qualitative and quantitative analyses with good skill to identify metabolites [46]. Mass spectrometers act by ion formation, which entails the separation of ions according to their mass-to-charge (m/z) ratio, and the detection of separated ions [9,47]. MS is rapidly gaining interest in metabolomics, though it is more often associated with other techniques, such as chromatography [48]. In fact, MS, which was widely developed over the last decades, holds a distinguished position in the field of determination, quantification and separation of mixtures of compounds. Recent advances in MS-based metabolomics produced the potential to quantify the levels of hundreds of metabolites that are intermediate or final products of cellular processes [49]. Thanks to its high sensitivity, selectivity and wide range of covered metabolites, MS has become the most widely used technique in metabolomics studies [2]. Another advantage of MS is derived from its reproducible quantitative analysis and its power to analyse samples with extremely high molecular complexity [50,51]. The objectives of developing MS for metabolomics range from the structural characterization of important metabolites to the detection of metabolite variations [52]. Moreover, MS can be applied to analyse biological samples, either by direct-injection or following chromatographic separations [3,53,54]. Recent progress and improvements in mass accuracy and precision have drastically increased the range of metabolites that can be analysed by MS, and have enhanced the accuracy of compound identification [48]. Usually, both of the above mentioned methods are used in metabolomics studies in order to identify and quantify all metabolites of the biological system under analysis [55,56]. Additionally, NMR provides users a vision of intact molecules at the atomic level and enables the viewing not only of 1-H atoms, but also many other kinds of atoms (^13^C, ^15^N) or biologically reactive groups, including phosphate atoms (^31^P) [57,58,59,60]. In addition, this technique needs minimal or no sample preparation and it is non-destructive, unlike MS [9,61]. Jeremy Nicholson was a pioneer in the use of NMR spectroscopy in the toxicological field [62]. Therefore, it is suitable for studies of cell extracts, and for cell cultures and tissues in vitro or in vivo. Among other advantages, it is highly reproducable and has the ability to identify unknown metabolites [63,64]. Our metabolomics work exclusively used ^1^H-NMR for the analyses of marine samples, but other nuclides (i.e., ^13^C, ^31^P, ^15^N, ^19^F, and ^2^H) may provide more information about this set of metabolites. The main limits of this technique concern the spectral resolution and sensitivity, which can be improved by high intensity magnetic field [57,65]. Furthermore, as compared to LC-MS and GC-MS, ^1^H-NMR spectroscopy is almost 100 times less sensitive. This means that a typical ^1^H-NMR-based metabolomics study only gives back information on 50–200 identified metabolites with concentrations >1 μM, while a typical LC-MS study can render information on more than 1000 identified metabolites with concentrations of >10 to 100 nM [57].

## 3. Metabolomics Applied to Marine Organisms in Cancer Studies

Cancer is one of the deadliest human diseases, able to alter the metabolism of a cell [66]. It is well known that cancer metabolism is different from that of normal tissue, and an important hypothesis published in the 1950s by Otto Warburg suggested that cancer cells use anaerobic metabolism as a source of energy [67]. In fact, the best-studied aspect of cancer metabolism is the central carbon metabolism and the relationships between glycolysis, the tricarboxylic acid (TCA) cycle and the oxidative phosphorylation [68]. One assumption is that cancer mainly converts pyruvate to lactate, rather than fuelling the TCA cycle, even in aerobic conditions [69,70]. The exploration of the cancer metabolome is the best way to understand the phenotypic changes associated to biological functions. Thanks to the metabolomics approach, it is possible to identify a range of metabolites involved in the process of carcinogenesis [71]. Moreover, a metabolomics approach is used for the discovery of biomarkers, and consequently, to improve the diagnosis and prognosis of many cancers, such as colorectal, breast, gastric, pancreatic and liver cancer [72]. In metabolomics, there are several approaches: fingerprinting, footprinting, profiling, flux analysis and target analysis. The first approach includes the screening of all metabolites within a biological system. Footprinting (mainly related to in vitro cell system) investigates metabolites from the environment around the system under analysis and shows information about metabolic exchange. Profiling is used to identify chemical compounds, for example lipids, also using standards for analyses. The fourth approach, called “flux analysis”, is the detection of one compound, usually isotope-labelled carbon, through a specific pathway or set of pathways, to determine the destiny of the compound. Target analysis provides a comparison of one or a few target metabolites, whose concentrations can change depending on the environmental conditions [72,73].

According to an analysis of literature, in the last years, the marine environment has shown to be the most promising source of bioactive compounds against cancer. In particular, sponges and algae are likely to be the marine organisms from which the largest number of natural compounds with antiproliferative activity could be isolated. During the past 50 years, sponges are considered a gold mine for the discovery of bioactive natural products [74,75]. In fact, the first marine-derived anticancer compound, the cytarabine or Ara-C, which was approved in 1969 and is still used to treat acute myelocytic leukemia and non-Hodgkin’s lymphoma, was isolated from the Caribbean sponge *Tethya crypta* [76]. In 2010 another anticancer agent, Eribulin, was isolated from the sponge *Halichondria okadai* [77], developed from the polyether metabolite halichondrin B and commercialized as Halaven [78]. Algae, especially microalgae, have the same importance as a source of natural products, being easily cultivated in large-scale closed ensuring a theoretically limitless supply of biomass. Bioactive compounds of algal origin can be sourced directly from primary metabolism (e.g., proteins, fatty acids, vitamins and pigments) or can be synthesized from secondary metabolism [79]. Potent sunscreens against ultraviolet (UV)-induced cell damage were isolated from Spirulina, Chlorella and Dunaliella [80,81,82,83], as well as antioxidant carotenoids (astaxanthin, lutein, zeaxanthin, canthaxanthin and b-carotene) from *Dunaliella salina* [84,85,86,87,88].

**Figure 4:**
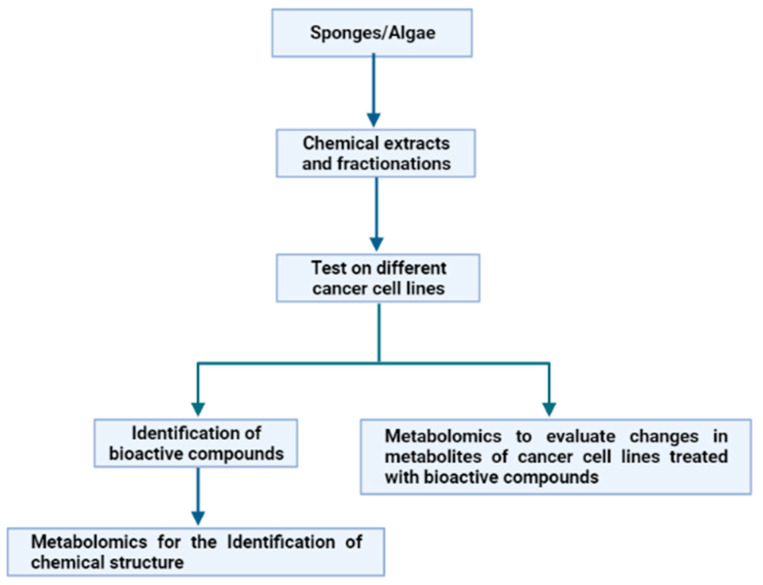
Experimental workflow of the two different applications of metabolomics to the study of marine extracts for anticancer applications.

In the frame of evolutive processes, several marine organisms, such as macro- and microalgae, sponges and fishes, developed appropriate defence mechanisms. They are based on the use of a variety of natural weapons, i.e., molecules that allow them to survive in a hostile environment characterized by stressful conditions. This is due to variable salinity, pressure, temperature and light, as well as to the need to avoid microbial and/or viral attacks [19]. These compounds, playing key ecological roles, are characterized by specific biological and potential biotechnological activities (anti-cancer, anti-inflammatory, anti-oxidant, anti-microbial, anti-hypertensive) worth explotation for pharmacological purposes. Sponges and algae represent promising resources for cancer treatment [20,21]. Consequently, our attention was focused on these two groups of marine organisms, in order to show how metabolomics have, in the past few years, aided in the exploitation of these organisms for several applications against cancer. In particular, we highlighted how metabolomics has been used with two different approaches, starting from the chemical extracts from sponges and algae (Figure 4).

The first approach involves the use of bioassay-guided fractionation of chemical extracts from the marine organisms under analysis on different cancer cell lines, and, once an active fraction has been identified, metabolomics approaches are applied to elucidate the chemical structure of the potentially bioactive compounds. Otherwise, in the second approach, the bioassay-guided fractionation of chemical extracts is also performed. However, once a cancer cell line on which the active fractions have antiproliferative effects has been identified, metabolomics approaches are applied to define the changes in metabolites of treated cells. To date, the data reported in literature (see below) demonstrated that the first approach is the most used and fewer applications have been reported on the second approach, because, at the moment, the second approach represents a novelty in the pharmacological field.

### 3.1. Structure Elucidation of Bioactive Molecules from Sponges

**Figure 5:**
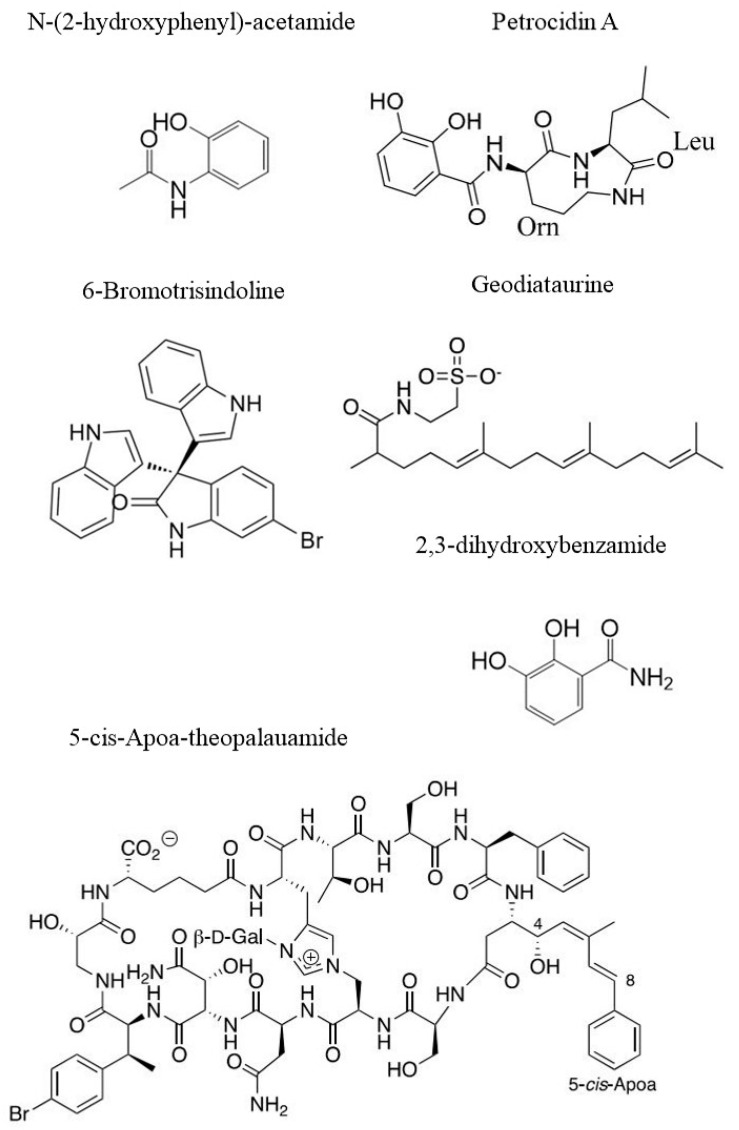
Examples of chemical structures of natural compounds from sponges: *N*-(2-hydroxyphenyl)-acetamide [108], Petrocidin A and 2,3-Dihydroxybenzamide [105], 6-Bromotrisindoline [94], Geodiataurine [98] and 5-*cis*-Apoa-theopalauamide [100].

Marine sponges (phylum Porifera) have been largely demonstrated to be one of the richest sources of exclusive secondary metabolites with relevant bioactivity, by means of bioassays [89,90]. Many scientists are trying to districate the complex network of causes and factors influencing the appearance of neoplastic cells [91,92]. Metabolomics represents a powerful tool for seeking potentially new and sustainable bioactive compounds in different species of sponges. In fact, two compounds (Stylissamide A and Stylissoside A) from the marine sponge *Stylissa carteri* sampled in the Red Sea, were extracted and analysed using LC-MS and ^1^H-NMR. Further, these compounds were tested on two cancer cell lines. The IC_50_ values for Stylissamide A on MCF7 (breast cancer) and on HepG2 (liver cancer) were of 21.1 µM and 36.8 µM, respectively, while the IC_50_ values for Stylissoside A were of 27.5 µM on MCF7 and 30.5 µM on HepG2 [93]. Similar metabolomic approaches (by LC-MS) led to the isolation and characterization of two compounds from another sponge species, *Callyspongia siphonella*. The molecules were characterized by using ^1^H-NMR and named 5-bromotrisindoline and 6-bromotrisindoline (see Figure 5 for chemical structure). In particular, 5-bromotrisindoline was effective against HT29 (colon carcinoma), OVCAR3 (ovarian carcinoma) and MM.1S (multiple myeloma) with IC_50_ of 8, 7 and 9 µM, respectively while 6-bromotrisindoline was effective against the same cancer cell lines with IC_50_ 12.5, 9 and 11 µM, respectively [94]. Some sponges living in extreme environmental conditions can also produce interesting compounds, which can be active against human cancer cell lines. This is the case of a specimen belonging to the sponge *Haliclona rosea*, collected in shallow water at a hydrothermal vent site. The extract of this sponge was analysed using the MS technique. This made possible the identification of several 3-alkyl-pyridine alkaloids (3-APA) responsible for the reduction of the 70–90% of cell viability of SKBR3 breast cancer cell line [95] at the concentration of 33 µg/mL in dichloromethane/methanol extracts. In particular, Cyclostellettamine P, one out of thirteen 3-APA compounds, was characterized, for the first time, by ion mobility mass spectrometry (IMS) in time-aligned parallel (TAP) fragmentation mode. This new technique permits the separation of ionic species as they drift through a gas phase under the influence of an electric field. Then, ions isolated in this way were subjected to subsequent fragmentation in the “trap” region of the IMS device [96]. LC-MS led to the identification of 20 compounds that could be responsible for the cytotoxic activity of the crude extract of *Coscinoderma* sp. on cancer cells. Crude extracts alone showed moderate activity, but were enhanced when the extract was encapsulated in liposomes. In this case, the IC_50_ values were notably lower compared to the positive control (Doxorubicin). The tested cell lines were HepG2 (IC_50_ = 2.2 µg/mL), MCF7 (IC_50_ = 4.1 µg/mL) and CaCo2 (colon cancer, IC_50_ = 1.7 µg/mL) [97]. A new compound named Geodiataurine (see Figure 5 for chemical structure) has been isolated from the marine sponge *Geodia macandrewii*, thanks to a complex combination of two techniques for the generation of metabolomics data: UHPLC and MS. The cytotoxic activity of this compound was tested against a melanoma cancer cell line (A2058) and showed weak cytotoxic activity (IC_50_ = 8.5 μM) [98]. Similarly, a sponge coming from the deep-sea Antarctic zone belonging to the species *Latrunculia biformis* caught the attention of Li et al. [99]. The authors revealed, thanks to ^1^H-NMR and MS analyses, the presence of known and unknown compounds. However, only three of them showed cytotoxic activity on the HCT-116 colon cancer cell line. The first tested compound was already known: (−)-discorhabdin L, and it showed IC_50_ equal to 0.33 µg/mL. The other two interesting compounds were new. They are two new discorhabdin analogs, i.e., (−)-1-acetyl-discorhabdin L and (+)-1-octacosatrienoyl-discorhabdin L, exhibiting IC_50_ of 1.1 µg/mL and 25.6 µg/mL, respectively [99]. Metabolite analyses of the marine sponge *Theonella swinhoei* performed by MS and ^1^H-NMR showed the presence of theonellamides. In particular, Theopalauamide was an already known compound whose cytotoxic activity was evaluated on the HTC-116 colon carcinoma cell line (IC_50_ = 2.8 µM), while two new compounds (5-*cis*-Apoa-theopalauamide reported in Figure 5 and theonellamide K) exhibited a cytotoxic activity on the same cell line, with IC_50_ of 21.8 and 3.5 µM, respectively [100].

We should consider, however, that sponges are holobionts that live in symbiosis with many bacterial species. These latter can represent up to 35% of the sponge’s weight [101]. Most of the symbiotic bacteria able to produce bioactive compounds are potential candidates for biotechnological applications [102,103,104]. From the marine sponge *Petrosia ficiformis* sampled in the Mediterranean Sea (Milos, Greece), a bacterial strain of Streptomyces sp. (SBT348) has been isolated. Thanks to a metabolomic approach performed through LC-MS analyses, it was possible to isolate a known compound, namely Petrocidin A (see Figure 5 for chemical strcture), which had cytotoxic effect towards HL-60 (human promyelocytic cell) and HT29 cell lines with IC_50_ 3.9 and 5.3 µg/mL, respectively. In addition, from the Streptomyces sp. (SBT348), a new compound whose structure has been elucidated (2,3-Dihydroxybenzamide, Figure 5) showed activity against the same cell lines with different IC_50_ values of 5.5 µg/mL and 3.8 µg/mL, respectively [105].

In another study, from samples of the Red Sea sponge *Callyspongia* sp., a strain of *Nocardiopsis* sp. (UR67) has been isolated, from which Nocardiotide A has been detected and characterized through MS analyses. Surprisingly, this compound showed activity against several different cancer cell lines, such as the CT26 (murine colon carcinoma, IC_50_ = 12 µM/mL), HeLa (human cervix carcinoma, IC_50_ = 11 µM/mL) and MM.1S (IC_50_ = 8 µM/mL) cancer cell lines [106]. Two bacterial strains belonging to Nocardia sp. (UR 86) and *Nocardiopsis* sp. (UR 92) were isolated from another sponge belonging to *Amphimedon* sp., coming from the Red Sea. These bacterial strains were cultured in different culture conditions and their crude extracts were analysed with an MS approach. The extract of *Nocardia* sp. (UR86) caused cellular inhibition of several cancer cell lines, such as HepG2, MCF7 and CaCo2, with IC_50_ values of 3.1 µg/mL, 3.9 µg/mL and 14.4 µg/mL, respectively. Similarly, extracts of *Nocardiopsis* sp. (UR92) were active against the same cancer cell lines with IC_50_ values of 3.7 µg/mL, 14.7 µg/mL and 14.3 µg/mL, respectively [107]. A newer approach is present in the research recently conducted by Hifnawy et al. [108]. They co-cultured two Actinomycetes to stimulate them to produce metabolites that would not have been produced if the two strains were cultured separately. These two actinomycetes were isolated from two sponges: *Micromonospora* sp. was isolated from *Callyspongia* sp., while *Actinokineospora* sp. was isolated from *Spheciospongia vagabunda*. Several compounds were detected using LC–MS metabolomics analyses, but only one was noteworthy in terms of its cytotoxic effect: the *N*-(2-hydroxyphenyl)-acetamide (see Figure 5). This was active against several cancer cell lines, such as HCT116 (colorectal carcinoma), HePG-2 (hepatocellular carcinoma) and MCF7 (mammary gland), with IC_50_ values ranging from 10 to 36 µM [108]. Extracts/compounds from sponges and their cytotoxic activities are summarized in Table 1.

### 3.2. Structure Elucidation of Bioactive Molecules from Algae

Algae are a complex and heterogeneous group of photosynthetic organisms, showing an extraordinary biological diversity represented by more than 166,000 species [109]. It is convenient to divide them into micro- (unicellular) and macro- (multicellular) algae according to their structure, evolution, ecological properties and sizes. These organisms produce and store a huge variety of metabolites, which include biologically active compounds (e.g., pigments, proteins and polysaccharides, antioxidants and polyunsaturated fatty acids), and several secondary metabolites produced in response to the pressures received in a wide range of environments, characterized by different conditions of temperature, light and salinity [110], among others. Bioactive molecules extracted both from microalgae and macroalgae show cytotoxic, antiviral and anti-inflammatory effects, with high potential for use in various medical fields. Some of those compounds are effective as therapeutic agents against cancer, showing high specificity for target molecules [111,112]. Moreover, seaweeds represent an excellent source of bioactive compounds because they are easy to cultivate, allowing for the production of larger biomasses that can be used for industrial purposes. Among the edible species, having an historical importance as source of food for human consumption, there are the seaweeds belonging to the genus Ulva, which have a range of health-promoting bioactive components. In particular, ulvan, a polysaccharide contained in its cell walls, is mainly composed of sulfated rhamnose, uronic acids (glucuronic acid and iduronic acid) and xylose. It showed cytotoxic activity against cancer cells [113,114]. In fact, according to Than et al. [114], ulvan extracted from *Ulva lactuca* had strong effect at various concentrations (0.8, 4, 20 and 100 μg/mL) against HepG2, MCF7 and Hela cancer cell lines. They also assessed the value of IC_50_ on the above three cell lines, being 29.7 μg/mL, 25.1 μg/mL and 36.3 μg/mL, respectively. In conclusion, ulvan showed a significant cytotoxic activity in a dose-dependent manner and it can be developed as a promising cancer-fighting compound. In the same work, the researchers determined the fine structure of the ulvan, using ^1^H-NMR and MS methods. Similarly, Mofeed et al. [115] tested an organic extract of *U. lactuca* and *Ulva fasciata* on MCF7 and HTC-116 (colorectal carcinoma) cell lines at different concentrations (12.5, 25, 50 and 100 μg/mL), highlighting a significant dose-dependent response after 48 h of exposure. They also tested the extracts of three additional species of seaweeds, which, similarly, exhibited cytotoxic activity: two red algae, namely *Amphiroa anceps* and *Corallina mediterranea*, and the fucales *Sargassum filipendula*. The extracts were analysed by means of GC-MS. Moreover, various researches have been carried out on seaweed belonging to order Fucales, such as *Fucus vesiculosus*. Geisen et al. [116] reported the inhibition of the cellular cycle in several cancer cell lines of pancreatic ductal adenocarcinoma (Panc1, PancTU1 and Panc89) and pancreatic adenosquamous carcinoma (Colo 357), testing fractions from a hydrophilic extract, after separation through HPLC. This effect seems related to the up-regulation of cell cycle inhibitors, showing an alteration of the expression levels of proteins and mRNA. Additionally, in the case of *F. vesiculosus*, Zenthoefer et al. [117] analysed the effect of six crude extracts, each of which was analysed by ^1^H-NMR spectroscopy techniques, revealing a characteristic fingerprint that was significantly correlated with the activity. In particular, the acetonic crude extract (FvT_A) showed the strongest activity against Panc89 and PancTu1, with an inhibitory rate of 80.3% and 82.6%, respectively. It is worth observing that the particular attention paid to some brown algae is due to the production and storage of a sulphated polysaccharide called Fucoidan, which is well known as a promising compound to be applied for cancer treatment. Among various species of brown algae, one of the best known is *Cladosiphon okamuranus*, an edible alga that is commonly cultured in Japan. This alga largely produces an accessory pigment named fucoxanthin, which is mainly metabolised and transformed into fucoxanthinol by the digestive enzymes of the gastrointestinal tracts of consumers. Both compounds are well known and studied because they exert an anti-proliferative effect [118]. Rokkaku et al. [119] extracted fucoxanthin and fucoxanthinol by the seaweed *C. okamuranus* using HPLC and MS. The results showed potentially anti-osteosarcoma properties, which appear to be at least partially attributable to the inhibition of Akt and AP-1 signal pathways in human and mouse osteosarcoma cancer cell lines (Saos-2, MNNG/HOS, 143B and LM8). In addition, various sulphated compounds extracted from algal biomasses have aroused interest for their biotechnological applications. In particular, Shao et al. [120] demonstrated the activity of three sulphated polysaccharides extracted by *U. fasciata* (UFP), *Gloiopeltis furcata* (GFP) and *Sargassum henslouianum* (SHP). The polysaccharides were extracted after ultrasonic disruption applying Radial Flow Chromatography (RFC) separation and then tested on MKN45 (gastric cancer) and DLD (intestinal cancer) cell lines. After incubating with those three extracts for 24 h at different concentrations (from 0.125 to 1.00 mg/mL), the inhibitive effects on MKN45 cancer cells were observed. In particular, SHP exhibited the strongest cytotoxic effect, with a growth inhibition percentage of almost 50% at the concentration of 1.00 mg/mL. In contrast, all samples showed low percentages of growth inhibition on DLD cancer cells, at all concentrations.

After the aforementioned works, dealing with various macroalgae, hereafter we explore studies focused on the use of microalgae as a source of anti-cancer compounds. The study of Abreu et al. [121] investigated the effectiveness of ^1^H-NMR and MS metabolomic approaches to record the variation of metabolites naturally present in the dinoflagellate *Amphidinium carterae* in a long-term culture. Specifically, among other compounds of interest, as fatty acids and carotenoids, they focused on the amphidinol family and their content variation in relationship to different levels of daily irradiance and nutrients in anf/2 medium. Three concentrations of methanolic extract (10, 30, and 100 μg/mL) were tested on four cancer cell lines: namely A549, HT29, MDA-MB-231 and PSN-1, showing a high antiproliferative activity against all four tumor cell lines (80%). Similarly, other studies investigated the chemical composition of the extracts of various microalgae along with their anticancer effects. Arslan et al. [122] analysed a crude extract of *Isochrysis galbana* through ^1^H-NMR and GC-MS and tested its cytotoxic effect on four cell lines: chronic myelogenous leukemia K562, human acute T lymphoblastic leukaemia MOLT-4, human Caucasian histiocytic lymphoma U937, Caucasian promyelocytic leukemia HL60 and human Burkitt′s lymphoma Raji cancer cells, showing the highest cytotoxicity (about 24.07%) at a concentration of 500 μg/mL against Raji cells. An interesting approach to the assay of natural products against cancer cell lines was reported by Karakas et al. [123]. They tested crude extracts of two microalgae (*Chlorella protothecoides* and *Nannochloropsis oculata*) against A172 (brain glioblastoma) and HCT116 cell lines. Assays on cells were carried out, testing not only the crude extract at increasing concentrations (25, 50, 100 μg/mL), but also three micro- and nano-particles loaded with the extracts, represented by PVA: Chitosan solution (PCH) and PVA: NaAlg solution (PNA) (produced through electrospraying techniques), and NPAL (obtained using the microemulsion method). The particles PCH and PNA were prepared by loading with concentrations of 70, 35 and 17.5 μg/mL, while NPAL particles obtained by the microemulsion method were loaded with 50, 25 and 12.5 μg/mL. Each particle was preliminarily tested on HUVEC cell lines (non-cancerous cells) in order to ensure absence of cytotoxicity against normal cells. Crude extracts were analysed with GC-MS. The results of the assay showed cytotoxic effects of both microalgal extracts and encapsulated microalgal extracts on two cancer cell lines while they did not have cytotoxic effects on healthy cells. This study showed that microalgal extracts have cytotoxic effects on cancer cells and did not lose their cytotoxic effects after encapsulation. In a similar study, Hussein et al. [124] analyzed the effects of crude extracts of *Tetraselmis suecica* in conjunction with an innovative compound, through the application of silver nanoparticles adopted as a carrier, against MCF7, mammary carcinoma 4 T1 and normal Vero cell-lines. The cytotoxicity assays were carried out by separately testing the crude extract of the microalgae, the silver nanoparticles AgNPs. In conclusion, the co-application of the two. *T. suecica* single application only showed the IC_50_ of 46.77 μg/mL on MCF7 and 83.17 μg/mL on 4 T1 cells. The AgNPs single application displayed the highest cytotoxicity according to a dose-dependent pathway after 72 h treatments with an IC_50_ of 5.3 μg/mL on MCF7, 17.78 μg/mL on 4 T1, and 25.11 μg/mL on Vero cells. Besides, the AgNPs-*T. suecica* co-application reached the IC_50_ of 6.60 μg/mL and 53.7 μg/mL, respectively, while the *T. suecica*-CHL single application only showed the IC_50_ of 46.77 μg/mL and 83.17 μg/mL against MCF7 and 4 T1, respectively. Moreover, they analysed the crude extract of *T. suecica* through GC-MS. In another study, Hussein et al. [125] demonstrated that the highest cytotoxic activity against MCF7 cells was exhibited by the synergic application of Tamoxifen (TMX, anti-estrogen drug) and *Nannochloropsis oculata*’s water extract with IC_50_ of 15.8 μg/mL, TMX-*T. suecica*’s ethanolic extract with IC_50_ of 16.9 μg/mL, TMX-*Chlorella* sp.’s chloroform extract with IC50 of 13.4 μg/mL, while TMX-*N. oculata*’s chloroform extract, TMX-*T. suecica*’s ethanolic extract and TMX-*Chrolella* sp.’s ethanolic extract showed cytotoxic effect against 4 T1 cells with IC_50_ of 15.4, 13.8 and 16.9 μg/mL, respectively. Moreover, the synergistic application of TMX-algae’s extracts maintains an antiproliferative effect on cancer cell lines and reduced the toxicity on normal Vero cells. In addition, after ^1^H-NMR analyses, isoleucine was found only in the ethanolic extract of *Chlorella* sp., glycerol only in the ethanolic extract of *T. suecica* and in the chloroform extract of *Chlorella* sp., while xanthine was found only in the chloroform extract of *Chlorella* sp. These metabolites can help reduce the toxicity of non-cancerous Vero cells. Fayyad et al. [126] tested various concentrations of hot methanolic extracts of *Spirulina platensis* to identify the most active chemical compounds and also to check the cytotoxic effect on cancer cell lines L20 B (mice intestine carcinoma) and MCF7 (breast cancer) after 24 h and 48 h exposure. GC mass analysis showed that the active chemical compounds in the extracts contained alkaloids, terpenes, phenols, resins, saponines, flavones, steroids, proteins and amino acids. Moreover, this extract exhibited the highest growth inhibition by testing the 12.5 mg/mL concentration against L20 B (32.5%) and against MCF7 (71.5%). These percentages increased after 48 h application (35.5% against L20 B and 78% against MCF7). Other studies, rather than testing the entire extract from microalgae, worked on different fractions obtained with distinct methods. El-Baz et al. [127] extracted carotenoid and polar fractions from both *Haematococcus pluvialis* and *Dunaliella salina* and tested them on HePG2, MCF7, HCT116, and A549 cancer cell lines. Moreover, carotenoids of *H. pluvialis* identified using LC-MS showed high cytotoxic activity against the HCT116 (100% inhibition at 0.1 mg/mL) and mild activity against both MCF7 and HePG2 lines, while the carotenoid-rich fraction of *D. salina* showed moderate cytotoxic activity on the MCF7 and HePG2 cancer cell lines.

Savio et al. [128] obtained hydrophilic and lipophilic fractions from the diatoms *Phaeodactylum tricornutum* and *Staurosirella pinnata* that were analysed with ^1^H-NMR. They performed a bioactivity assay on human immortalised keratinocytes HaCaT and human melanoma CHL-1 cell lines in a 24 h dose-response test with different concentrations (0.2, 0.4, 0.8, 1.6, 3.2 and 10.0 mg/mL). The most important result showed that *S. pinnata* extract had an important dose-dependent effect on CHL-1. Extracts and compounds from the macro- and micro-algae analysed in this section are shown in Table 2.

### 3.3. Metabolite Changes in Treated Cancer Cell Lines

As mentioned above, a few studies report analyses of metabolite changes after treatment of cancer cells with extracts/molecules isolated from sponges or algae. An interesting method to study cytotoxic activity is to provide a metabolic profiling of the treated cells to understand at which level the compound is interacting. This was the aim of the research conducted by Costantini et al. [129]. The studied sponge was *Geodia cydonium* and it was tested on three breast cancer cell lines, MCF7, MDA-MB-231 and MDA-MB-468. An organic extract of this sponge had a cytotoxic effect with IC_50_ of 37, 44 and 70 μg/mL, respectively, on three cancer cell lines analysed, whereas it was inactive on normal breast cells (MCF-10A). After the treatment, the metabolomic profile was studied through ^1^H-NMR spectra. Interestingly, the active fraction was able to interfere with the glycolysis, lipid and amino acid metabolism of the tumor cells, enabling them to support their bioenergetic and macromolecular synthesis. In particular, the proton resonances related to the metabolites identified in three breast cancer cells were studied. The spectral region from 0.5 to 3 ppm showed the presence of signals from alanine, arginine, aspartate, glutamate, glutamine, isoleucine, lactate, leucine, lipids, lysine, proline, threonine and valine. The spectral region from 3 to 5.5 ppm was mainly composed of signals corresponding to choline, -glucose, -glucose, glycine, glycero-phosphocholine and phosphocholine. The 5.5–8.5 ppm region contained the resonances of histidine, phenylalanine and tyrosine. They also showed an increasing level of lactate after treatment in all three cell lines, and a decrease of α-glucose, β-glucose, choline, glycerophosphocholine, glutamine, glutamate and lipids. Other metabolites were also reduced: proline in MCF-7 cells, threonine in MDA-MB231 and asparagine and lysine in MDA-MB468 cells, whereas glycine was reduced in both MDA-MB231 and MDA-MB468 cells.

A decrease of pro-inflammatory cytokines (VEGF, CXCL10 and IL-8) levels was detected, as well, along with an increase of anti-inflammatory cytokines levels (IL-4 and IL-10). Finally, the chemical entities present in this fraction were analyzed by liquid chromatography high-resolution mass spectrometry combined with molecular networking.

An additional interesting approach to the brown macroalga *F. vesiculosus* was performed by Heavisides et al. [130], who investigated seasonal variations in the metabolome of this inhabitant of the Baltic Sea and its potential relation to the bioactivity profile. The authors developed an optimised protocol in order to extract algal biomass monthly for a full year, employing UHPLC-MS for untargeted metabolome analysis. Simultaneously, these crude extracts were screened to evaluate their cytotoxic activity on A-549 (lung adenocarcinoma), MB-231 (breast carcinoma) and Panc1 cell lines, demonstrating that an organic extract of the alga sampled in June exhibited caspase 3/7 activity of 2.2 for the Panc1 cancer cell line. The extracts were also tested for their general toxicity on human keratinocyte (HaCaT) cell lines, against which no activity was recorded, implying a lack of general toxicity for normal cells. In addition, results of this study showed the variation of many compounds according to the sampling month; for example, the highest content in phlorotannin was recorded during the summer period. Literature analyses reference the detection of the antiproliferative effect of *F. vesiculosus* due to phlorotannins [117], fucoidan [131] and fucoxanthin [132]. This study highlighted the importance of the impact of the sampling season on the bioactivity and metabolite profile.

## 4. Conclusions

Among the “omics” platforms, metabolomics has a great power to aid cancer research, thanks to the possibility for rapid analysis of tissue or biofluid samples. In fact, by coupling metabolite profiling and organism biology, it is possible to provide significant impacts for the discovery of new compounds and define their possible biotechnological applications for blocking the progression of cancer. Metabolic profiling is usually referred to a quantitative study of a group of metabolites that is associated with a given pathway. As demonstrated by the data reported in this review, metabolomics has been extensively used in the last years, with the aim to isolate bioactive compounds from marine organisms (mainly from sponges and algae) against cancer, defining their chemical structures. A few examples, in contrast, may be reported on the use of a metabolomics approach to study how treatment with chemical extracts from sponges and algae could induce metabolite changes in treated cancer cell lines. For this reason, we think that this is a strength of our review, pushing the scientific world to invest in research projects aimed to test marine natural products in metabolomic studies. In fact, we think that this represents a very important application for cancer research, helping in the understanding of changes in the metabolic pathways induced by natural compounds from the sea. This is possible because of a high diversity of marine natural products, which represent promising opportunities for drug discovery and the development of marine biotechnologies. In addition, high-throughput techniques, such as metabolomics, are extremely useful for rapidly exploring the chemical diversity of marine environments with respect to the classical approaches, being also able to detect metabolites when present at low concentrations. Metabolite identification remains the main metabolomics bottleneck, and together with bioinformatic tools, such as molecular networks, it can lead to the annotation of unknown metabolites, leading to the discovery of new compounds. Furthermore, understanding the ecological and biological factors that contribute to the production of a certain metabolite can be extremely useful for selecting and optimizing the extraction of bioactive compounds, enhancing their yields and elucidating gene clusters associated with the biosynthetic pathways to which they belong.

## Figures and Tables

**Table 1:** Sponge species, compound/extract, cell lines tested, metabolomics techniques and corresponding reference are reported.

Sponge	Compound/Extract	Cell Lines	Metabolomics	Reference
*S. carteri*	Stylissamide A and Stylissoside A	MCF7 and HepG2	LC-MS	[93]
*C. siphonella*	5-bromotrisindoline and 6-bromotrisindoline	HT29, OVCAR3 and MM.1S	LC-MS	[94]
*H. rosea*	3-alkyl pyridine alkaloids	SKBR3	MS	[95]
*Coscinoderma* sp.	Crude extract	HepG2, MCF7 and Caco2	LC-MS	[97]
*G. macandrewii*	Geodiataurine	A2058	UHPLC and MS	[98]
*L. biformis*	(−)-discorhabdin L, (−)-1-acetyl-discorhabdin L and (+)-1-octacosatrienoyl-discorhabdin L	HCT116	1H-NMR and MS	[99]
*T. swinhoei*	Theopalauamide, 5-*cis*-Apoa-theopalauamide and Theonellamide K	HCT116	1D and 2D NMR; MS	[100]
*P. ficiformis*	Petrocidin A and 2,3-Dihydroxybenzamide	HL60 and HT29	LC-MS	[105]
*Callyspongia* sp.	Nocardiotide A	CT26, HeLa and MM.1S	MS	[106]
*Amphimedon* sp.	Crude extract	HepG2, MCF7 and Caco2	MS	[107]
*Callyspongia* sp. and *S. vagabunda*	*N*-(2-hydroxyphenyl)-acetamide	HCT116, HepG2 and MCF7	LC–MS	[108]

**Table 2:** Algal species, compound or extract, cell lines tested, metabolomics techniques and corresponding reference are reported.

Algae	Compound/Extract	Cell Lines	Metabolomics	Reference
*U. lactuca*	Ulvan	HepG2, MCF7 and Hela	^1^H-NMR-MS	[114]
*U. fasciata, U. lactuca, A. anceps, C. mediterranea* and *S. filipendula*	Organic extract	MCF7 and HTC116	GC-MS	[115]
*F. vesiculosus*	Hydrophilic extract	Panc1, PancTU1, Panc89 and Colo 357	HPLC	[116]
*F. vesiculosus*	Crude extracts	Panc89 and PancTU1	^1^H-NMR	[117]
*C. okamuranus*	Fucoxanthin and fucoxanthinol	Saos-2, MNNG/HOS, 143 B and LM8	HPLC-MS	[119]
*U. fasciata, G. furcata* and *S. henslouianum*	Sulphated polysaccharides	MKN45 and DLD	RFC	[120]
*A. carterae*	Methanolic extract	A549, HT29, MDA-MB-231 and PSN-1	^1^H-NMR and MS	[121]
*I. galbana*	Crude extract	K562, MOLT-4, U937, HL60 and Burkitt′s lymphoma	^1^H-NMR, GC-MS	[122]
*C. protothecoides* and *N. oculata*	Crude extract	A172 and HTC116	GC-MS	[123]
*T. suecica*	Crude extract	A172 and HTC116	GC-MS	[124]
*N. oculata, T. suecica* and *Chlorella* sp.	Water, ethanolic and methanolic extracts	MCF7 and 4 T1	^1^H-NMR	[125]
*S. platensis*	Methanolic extract	L20 B and MCF7	GC-MS	[126]
*H. pluvialis* and *D. salina*	Carotenoid fractions and polar fractions	HepG2, MCF7, HCT116 and A549	LC-MS	[127]
*P. tricornutum* and *S. pinnata*	Hydrophilic and lipophilic fractions	CHL-1	^1^H-NMR	[128]
